# 4′-Bromo­butyl *ent*-16-oxobeyeran-19-oate

**DOI:** 10.1107/S1600536810005167

**Published:** 2010-02-13

**Authors:** Junqing Chen, Xiaoming Zha

**Affiliations:** a School of Chemistry and Chemical Engineering, Institute of Pharmaceutical Engineering, Southeast University, Nanjing 210096, People’s Republic of China; b Jiangsu Center for Drug Screening, China Pharmaceutical University, 24, Tongjiaxiang, Nanjing 210009, People’s Republic of China

## Abstract

The title compound, C_24_H_37_BrO_3_, is a tetra­cyclic diterpenoid with a beyerane skeleton, synthesized by esterification of isosteviol. It comprises a fused four-ring system *A*/*B*/*C*/*D*. Rings *A* and *B* have a chair conformation, whereas ring *C* is an unsymmetrical distorted chair; the remaining five-membered ring *D* adopts an envelope conformation. The stereochemistry of the *A*/*B* and *B*/*C* ring junctions are *trans, *while the *C/D* junction is *cis.*

## Related literature

For the pharmacological activity of isosteviol, see: Liu *et al.* (2001 ▶); Mizushina *et al.* (2005 ▶); Wong *et al.* (2004 ▶); Xu *et al.* (2007 ▶). For ring conformations, see: Cremer & Pople (1975 ▶). For the synthesis of isosteviol derivates *via* esterification and bromination, see: Cai *et al.* (2009 ▶); Chen (2010 ▶).
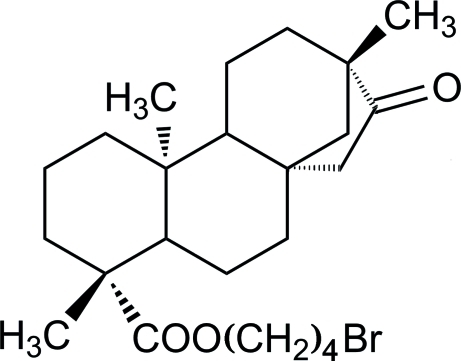


## Experimental

### 

#### Crystal data

C_24_H_37_BrO_3_
*M* *_r_* = 453.45
Orthorhombic, 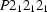

*a* = 7.4335 (10) Å
*b* = 9.7732 (14) Å
*c* = 30.920 (4) Å
*V* = 2246.3 (5) Å^3^
*Z* = 4
Mo *K*α radiation
μ = 1.85 mm^−1^
*T* = 298 K
0.45 × 0.43 × 0.37 mm

#### Data collection

Bruker SMART CCD area-detector diffractometer
Absorption correction: multi-scan (*SADABS*; Bruker, 1999 ▶) *T*_min_ = 0.490, *T*_max_ = 0.548
11802 measured reflections
3955 independent reflections
3041 reflections with *I* > 2/s(*I*)
*R*_int_ = 0.052

#### Refinement

*R*[*F* ^2^ > 2σ(*F* ^2^)] = 0.042
*wR*(*F* ^2^) = 0.108
*S* = 1.00
3955 reflections
256 parameters
H-atom parameters constrained
Δρ_max_ = 0.64 e Å^−3^
Δρ_min_ = −0.25 e Å^−3^
Absolute structure: Flack (1983 ▶), 1657 Friedel pairs
Flack parameter: 0.065 (11)

### 

Data collection: *SMART* (Bruker, 1999 ▶); cell refinement: *SAINT* (Bruker, 1999 ▶); data reduction: *SAINT*; program(s) used to solve structure: *SHELXS97* (Sheldrick, 2008 ▶); program(s) used to refine structure: *SHELXL97* (Sheldrick, 2008 ▶); molecular graphics: *SHELXTL* (Sheldrick, 2008 ▶); software used to prepare material for publication: *SHELXTL* and *PLATON* (Spek, 2009 ▶).

## Supplementary Material

Crystal structure: contains datablocks I, New_Global_Publ_Block. DOI: 10.1107/S1600536810005167/bg2330sup1.cif

Structure factors: contains datablocks I. DOI: 10.1107/S1600536810005167/bg2330Isup2.hkl

Additional supplementary materials:  crystallographic information; 3D view; checkCIF report

## Figures and Tables

**Fig. 1.**
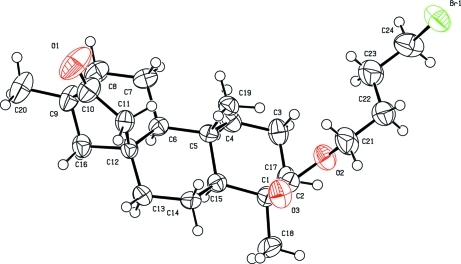
The molecular structure of the title compound with the atom-numbering scheme. Displacement ellipsoids are drawn at the 30% probability level.

## supplementary crystallographic information

### Comment

Isosteviol is a tetracyclic diterpenoid with a beyerane skeleton, which has good pharmacological activity against broad spectrum significant diseases including ischemia-reperfusion injury, hypertension, and cancer (Wong *et al.*, 2004; Liu, *et al.*, 2001; Xu, *et al.*, 2007; Mizushina *et al.*, 2005). The title compound was obtained by esterification of isosteviol. The molecule structure of (I) contains a fused four-ring system *A*/*B*/*C*/*D* (Fig. 1). The *A*/*B* ring and *B*/*C* junction are *trans*-fused, while *C*/*D* is *cis*-fused. Rings *A* and *B* adopt chair conformations (Puckering parameters as defined by Cremer & Pople, 1975: *Q* = 0.554 (4)/0.559 (4) Å, θ= 176.8 (4)/170.4 (4)° and φ= 68 (7)/83 (2)° , respectively), while ring *C* is in a distorted chair conformation with puckering amplitude *Q* = 0.647 (4) Å, θ= 18.1 (4)° φ=253.0 (13)°. The distortion may be attributed to the narrowing of the C9—C16—C12 bond angle to 104.2 (3)°. The five-membered ring *D* adopts an envelope conformation (puckering parameters *Q* = 0.456 (5)Å, φ = 140.7 (6) °) with atom C16 displaced from the C9/C10/C11/C12 plane by 0.297 (4) Å . The C17—C1—C2—C3 torsion angle of -74.8 (5)° describes the *β*-orientation of the 4'-bromobutyl ester group with respect to the ent-kaurane nucleus.

### Experimental

Isosteviol was obtained by hydrolysis of stevioside with 10% sulfuric acid at 95 °C for 7 h and recrystallization from ethanol gave colorless crystals of isosteviol in 80% yield. A mixture of 1,4-dibromobutane (2.4 ml, 20 mmol), K_2_CO_3_ (2.8 g, 20 mmol) and acetonitrile (20 ml) was heated to reflux. Isosteviol (3.2 g, 10 mmol) in 30 ml acetonitrile was added dropwise over 10 min, and the resulting mixture was stirred for 2 h further. The mixture was cooled to room temperature, and then distilled to one third volume under reduced pressure. The residue was poured into ice water, and the aqueous layer was extracted with CH_2_Cl_2_ (3 × 50 ml). The combined CH_2_Cl_2_ extracts were washed with water (1 × 50 ml) and brine (1 × 50 ml) respectively, and then dried with anhydrous Na_2_SO_4_. After the solvent was evaporated, the residue was purified by column chromatography on silica (petroleum ether/ethyl acetate = 18:1, v/v) to give the title compound (2.7 g, 60%). Crystals of the title compound suitable for X-ray diffraction were obtained by slow evaporation of ethanol solution at room temperature. m.p. 372-373 K; ^1^H NMR(300 MHz, CDCl_3_), δ_H_ ppm: 0.74(s, 3H), 0.90(s, 3H), 1.18(s, 3H), 0.96-2.01(m, 22H), 2.17-2.22(d, 1H, *J*=15.00 Hz), 2.49-2.56(dd, 1H, *J*=18.37, 3.57 Hz), 3.53-3.57(t, 2H, *J*=6.60 Hz), 4.00-4.14(m, 2H).

### Refinement

All H atoms were placed in geometrical positions and constrained to ride on their parent atoms with C–H distances in the range 0.96–0.98 Å, and included in the final cycles of refinement using a riding model, with *U*_iso_(H) = 1.5*U*_eq_(C) for methyl H and 1.2*U*_eq_(C) for other H atoms.

### Crystal data

**Table:**

C_24_H_37_BrO_3_	*D*_x_ = 1.341 Mg m^−^^3^
*M**_r_* = 453.45	Melting point = 372–373 K
Orthorhombic, *P*2_1_2_1_2_1_	Mo *K*α radiation, λ = 0.71073 Å
Hall symbol: P 2ac 2ab	Cell parameters from 3390 reflections
*a* = 7.4335 (10) Å	θ = 2.2–20.1°
*b* = 9.7732 (14) Å	µ = 1.85 mm^−^^1^
*c* = 30.920 (4) Å	*T* = 298 K
*V* = 2246.3 (5) Å^3^	Block, colourless
*Z* = 4	0.45 × 0.43 × 0.37 mm
*F*(000) = 960	

### Data collection

**Table:**

Bruker SMART CCD area-detector diffractometer	3955 independent reflections
Radiation source: fine-focus sealed tube	3041 reflections with *I* > 2/s(*I*)
graphite	*R*_int_ = 0.052
phi and ω scans	θ_max_ = 25.0°, θ_min_ = 2.2°
Absorption correction: multi-scan (*SADABS*; Bruker, 1999)	*h* = −8→8
*T*_min_ = 0.490, *T*_max_ = 0.548	*k* = −11→11
11802 measured reflections	*l* = −25→36

### Refinement

**Table:**

Refinement on *F*^2^	Secondary atom site location: difference Fourier map
Least-squares matrix: full	Hydrogen site location: inferred from neighbouring sites
*R*[*F*^2^ > 2σ(*F*^2^)] = 0.042	H-atom parameters constrained
*wR*(*F*^2^) = 0.108	*w* = 1/[σ^2^(*F*_o_^2^) + (0.0392*P*)^2^ + 0.9109*P*] where *P* = (*F*_o_^2^ + 2*F*_c_^2^)/3
*S* = 1.00	(Δ/σ)_max_ = 0.001
3955 reflections	Δρ_max_ = 0.64 e Å^−^^3^
256 parameters	Δρ_min_ = −0.25 e Å^−^^3^
0 restraints	Absolute structure: Flack (1983), 1657 Friedel pairs
Primary atom site location: structure-invariant direct methods	Flack parameter: 0.065 (11)

### Special details

**Table:**

Geometry. All esds (except the esd in the dihedral angle between two l.s. planes) are estimated using the full covariance matrix. The cell esds are taken into account individually in the estimation of esds in distances, angles and torsion angles; correlations between esds in cell parameters are only used when they are defined by crystal symmetry. An approximate (isotropic) treatment of cell esds is used for estimating esds involving l.s. planes.
Refinement. Refinement of *F*^2^ against ALL reflections. The weighted *R*-factor wR and goodness of fit *S* are based on *F*^2^, conventional *R*-factors *R* are based on *F*, with *F* set to zero for negative *F*^2^. The threshold expression of *F*^2^ > σ(*F*^2^) is used only for calculating *R*-factors(gt) etc. and is not relevant to the choice of reflections for refinement. *R*-factors based on *F*^2^ are statistically about twice as large as those based on *F*, and *R*- factors based on ALL data will be even larger.

### Fractional atomic coordinates and isotropic or equivalent isotropic displacement parameters (Å^2^)

**Table:**

	*x*	*y*	*z*	*U*_iso_*/*U*_eq_	
Br1	1.06095 (7)	0.65680 (5)	0.843138 (17)	0.06872 (19)	
O1	0.6425 (6)	1.2658 (4)	0.55233 (12)	0.0896 (14)	
O2	0.4910 (4)	0.6155 (3)	0.72346 (9)	0.0539 (8)	
O3	0.2689 (5)	0.7630 (3)	0.71082 (10)	0.0596 (8)	
C1	0.3384 (5)	0.5981 (4)	0.65535 (13)	0.0409 (9)	
C2	0.4651 (7)	0.4761 (4)	0.64746 (13)	0.0509 (11)	
H2A	0.4648	0.4187	0.6731	0.061*	
H2B	0.4174	0.4222	0.6238	0.061*	
C3	0.6590 (7)	0.5144 (4)	0.63695 (14)	0.0500 (11)	
H3A	0.7150	0.5539	0.6624	0.060*	
H3B	0.7254	0.4324	0.6293	0.060*	
C4	0.6691 (6)	0.6156 (4)	0.59994 (14)	0.0465 (10)	
H4A	0.6281	0.5710	0.5737	0.056*	
H4B	0.7937	0.6418	0.5957	0.056*	
C5	0.5556 (5)	0.7466 (4)	0.60725 (11)	0.0336 (8)	
C6	0.5564 (5)	0.8292 (4)	0.56375 (11)	0.0348 (8)	
H6	0.5287	0.7622	0.5411	0.042*	
C7	0.7413 (6)	0.8882 (4)	0.55177 (13)	0.0463 (11)	
H7A	0.7822	0.9468	0.5751	0.056*	
H7B	0.8265	0.8136	0.5490	0.056*	
C8	0.7401 (6)	0.9696 (5)	0.51008 (14)	0.0522 (11)	
H8A	0.7356	0.9063	0.4859	0.063*	
H8B	0.8519	1.0203	0.5080	0.063*	
C9	0.5820 (7)	1.0706 (4)	0.50609 (13)	0.0511 (11)	
C10	0.5754 (7)	1.1544 (5)	0.54781 (14)	0.0539 (11)	
C11	0.4664 (6)	1.0794 (4)	0.58069 (12)	0.0403 (10)	
H11A	0.3606	1.1320	0.5886	0.048*	
H11B	0.5369	1.0627	0.6065	0.048*	
C12	0.4113 (5)	0.9427 (4)	0.55920 (11)	0.0355 (9)	
C13	0.2273 (5)	0.8911 (4)	0.57262 (13)	0.0431 (10)	
H13A	0.1846	0.8260	0.5513	0.052*	
H13B	0.1437	0.9673	0.5732	0.052*	
C14	0.2291 (5)	0.8229 (4)	0.61678 (13)	0.0401 (9)	
H14A	0.1090	0.7917	0.6240	0.048*	
H14B	0.2667	0.8884	0.6385	0.048*	
C15	0.3585 (5)	0.7013 (4)	0.61649 (12)	0.0356 (9)	
H15	0.3237	0.6482	0.5909	0.043*	
C16	0.4094 (6)	0.9881 (4)	0.51133 (12)	0.0454 (10)	
H16A	0.4090	0.9094	0.4922	0.055*	
H16B	0.3046	1.0440	0.5052	0.055*	
C17	0.3620 (6)	0.6695 (5)	0.69846 (13)	0.0428 (10)	
C18	0.1425 (6)	0.5419 (5)	0.65644 (17)	0.0630 (13)	
H18A	0.1340	0.4703	0.6776	0.095*	
H18B	0.1115	0.5062	0.6285	0.095*	
H18C	0.0611	0.6145	0.6639	0.095*	
C19	0.6359 (5)	0.8274 (4)	0.64501 (12)	0.0404 (9)	
H19A	0.6488	0.7684	0.6696	0.061*	
H19B	0.5575	0.9021	0.6522	0.061*	
H19C	0.7516	0.8625	0.6368	0.061*	
C20	0.5970 (8)	1.1571 (5)	0.46522 (16)	0.0774 (16)	
H20A	0.5984	1.0984	0.4403	0.116*	
H20B	0.7061	1.2096	0.4661	0.116*	
H20C	0.4958	1.2179	0.4635	0.116*	
C21	0.5242 (7)	0.6883 (5)	0.76431 (14)	0.0647 (14)	
H21A	0.4163	0.6879	0.7820	0.078*	
H21B	0.5570	0.7826	0.7585	0.078*	
C22	0.6719 (6)	0.6183 (5)	0.78729 (15)	0.0587 (12)	
H22A	0.6383	0.5234	0.7917	0.070*	
H22B	0.6857	0.6599	0.8156	0.070*	
C23	0.8505 (7)	0.6223 (7)	0.76451 (16)	0.0757 (16)	
H23A	0.8386	0.5775	0.7367	0.091*	
H23B	0.8829	0.7170	0.7592	0.091*	
C24	0.9989 (7)	0.5548 (6)	0.78940 (17)	0.0716 (15)	
H24A	1.1049	0.5484	0.7712	0.086*	
H24B	0.9626	0.4625	0.7970	0.086*	

### Atomic displacement parameters (Å^2^)

**Table:**

	*U*^11^	*U*^22^	*U*^33^	*U*^12^	*U*^13^	*U*^23^
Br1	0.0692 (3)	0.0608 (3)	0.0761 (3)	0.0049 (3)	−0.0274 (3)	−0.0054 (3)
O1	0.135 (4)	0.054 (2)	0.080 (3)	−0.039 (2)	0.034 (2)	−0.0121 (19)
O2	0.060 (2)	0.0612 (19)	0.0409 (16)	0.0070 (15)	−0.0069 (14)	−0.0048 (14)
O3	0.070 (2)	0.062 (2)	0.0470 (18)	0.0160 (19)	0.0007 (16)	−0.0034 (16)
C1	0.046 (2)	0.040 (2)	0.037 (2)	−0.0072 (18)	−0.004 (2)	0.0044 (19)
C2	0.076 (3)	0.034 (2)	0.043 (3)	0.002 (2)	−0.008 (2)	0.0040 (18)
C3	0.062 (3)	0.041 (2)	0.047 (3)	0.016 (2)	−0.003 (2)	0.003 (2)
C4	0.043 (2)	0.050 (3)	0.047 (2)	0.011 (2)	−0.0043 (19)	−0.005 (2)
C5	0.032 (2)	0.0344 (19)	0.035 (2)	−0.0022 (19)	−0.0021 (18)	−0.0015 (16)
C6	0.037 (2)	0.0328 (18)	0.0346 (19)	−0.004 (2)	0.0010 (17)	−0.0038 (16)
C7	0.040 (2)	0.046 (2)	0.053 (3)	−0.0039 (19)	0.009 (2)	−0.006 (2)
C8	0.053 (3)	0.057 (3)	0.047 (3)	−0.006 (2)	0.015 (2)	−0.004 (2)
C9	0.073 (3)	0.043 (2)	0.037 (2)	−0.007 (2)	0.009 (2)	0.0028 (18)
C10	0.068 (3)	0.039 (2)	0.054 (3)	−0.006 (3)	0.010 (2)	−0.002 (2)
C11	0.048 (3)	0.034 (2)	0.039 (2)	0.0043 (19)	0.0012 (19)	0.0017 (17)
C12	0.041 (2)	0.038 (2)	0.0273 (19)	−0.0003 (18)	−0.0034 (17)	0.0009 (16)
C13	0.037 (2)	0.045 (2)	0.047 (2)	0.0042 (18)	−0.0059 (18)	0.007 (2)
C14	0.029 (2)	0.046 (2)	0.046 (2)	−0.0009 (19)	0.0018 (17)	0.004 (2)
C15	0.037 (2)	0.039 (2)	0.031 (2)	−0.0041 (17)	−0.0016 (17)	−0.0046 (16)
C16	0.057 (3)	0.045 (2)	0.035 (2)	−0.001 (2)	−0.006 (2)	0.0011 (18)
C17	0.044 (2)	0.044 (2)	0.041 (2)	−0.005 (2)	−0.0008 (18)	0.009 (2)
C18	0.061 (3)	0.069 (3)	0.059 (3)	−0.029 (2)	−0.005 (3)	0.009 (3)
C19	0.037 (2)	0.047 (2)	0.037 (2)	−0.0021 (18)	−0.0049 (16)	−0.003 (2)
C20	0.108 (5)	0.065 (3)	0.060 (3)	−0.013 (4)	0.020 (3)	0.018 (3)
C21	0.075 (3)	0.082 (4)	0.037 (2)	−0.001 (3)	−0.012 (2)	−0.006 (2)
C22	0.059 (3)	0.069 (3)	0.048 (3)	−0.004 (2)	−0.004 (2)	0.000 (2)
C23	0.064 (3)	0.111 (5)	0.052 (3)	−0.003 (3)	−0.001 (2)	0.009 (3)
C24	0.056 (3)	0.091 (4)	0.068 (3)	−0.004 (3)	−0.004 (3)	−0.016 (3)

### Geometric parameters (Å, °)

**Table:**

Br1—C24	1.992 (5)	C11—C12	1.547 (5)
O1—C10	1.206 (5)	C11—H11A	0.9700
O2—C17	1.340 (5)	C11—H11B	0.9700
O2—C21	1.470 (5)	C12—C13	1.516 (6)
O3—C17	1.208 (5)	C12—C16	1.545 (5)
C1—C17	1.515 (6)	C13—C14	1.519 (5)
C1—C2	1.539 (6)	C13—H13A	0.9700
C1—C18	1.557 (6)	C13—H13B	0.9700
C1—C15	1.576 (5)	C14—C15	1.529 (5)
C2—C3	1.524 (6)	C14—H14A	0.9700
C2—H2A	0.9700	C14—H14B	0.9700
C2—H2B	0.9700	C15—H15	0.9800
C3—C4	1.515 (6)	C16—H16A	0.9700
C3—H3A	0.9700	C16—H16B	0.9700
C3—H3B	0.9700	C18—H18A	0.9600
C4—C5	1.549 (5)	C18—H18B	0.9600
C4—H4A	0.9700	C18—H18C	0.9600
C4—H4B	0.9700	C19—H19A	0.9600
C5—C19	1.531 (5)	C19—H19B	0.9600
C5—C15	1.557 (5)	C19—H19C	0.9600
C5—C6	1.569 (5)	C20—H20A	0.9600
C6—C7	1.535 (6)	C20—H20B	0.9600
C6—C12	1.554 (5)	C20—H20C	0.9600
C6—H6	0.9800	C21—C22	1.476 (7)
C7—C8	1.515 (6)	C21—H21A	0.9700
C7—H7A	0.9700	C21—H21B	0.9700
C7—H7B	0.9700	C22—C23	1.503 (7)
C8—C9	1.540 (7)	C22—H22A	0.9700
C8—H8A	0.9700	C22—H22B	0.9700
C8—H8B	0.9700	C23—C24	1.499 (7)
C9—C16	1.524 (6)	C23—H23A	0.9700
C9—C20	1.525 (6)	C23—H23B	0.9700
C9—C10	1.529 (6)	C24—H24A	0.9700
C10—C11	1.492 (6)	C24—H24B	0.9700
			
C17—O2—C21	115.1 (3)	C12—C13—C14	112.6 (3)
C17—C1—C2	115.2 (3)	C12—C13—H13A	109.1
C17—C1—C18	104.6 (4)	C14—C13—H13A	109.1
C2—C1—C18	107.6 (3)	C12—C13—H13B	109.1
C17—C1—C15	111.4 (3)	C14—C13—H13B	109.1
C2—C1—C15	108.5 (3)	H13A—C13—H13B	107.8
C18—C1—C15	109.3 (3)	C13—C14—C15	110.0 (3)
C3—C2—C1	115.0 (3)	C13—C14—H14A	109.7
C3—C2—H2A	108.5	C15—C14—H14A	109.7
C1—C2—H2A	108.5	C13—C14—H14B	109.7
C3—C2—H2B	108.5	C15—C14—H14B	109.7
C1—C2—H2B	108.5	H14A—C14—H14B	108.2
H2A—C2—H2B	107.5	C14—C15—C5	111.8 (3)
C4—C3—C2	111.6 (4)	C14—C15—C1	115.7 (3)
C4—C3—H3A	109.3	C5—C15—C1	114.3 (3)
C2—C3—H3A	109.3	C14—C15—H15	104.5
C4—C3—H3B	109.3	C5—C15—H15	104.5
C2—C3—H3B	109.3	C1—C15—H15	104.5
H3A—C3—H3B	108.0	C9—C16—C12	104.2 (3)
C3—C4—C5	113.8 (3)	C9—C16—H16A	110.9
C3—C4—H4A	108.8	C12—C16—H16A	110.9
C5—C4—H4A	108.8	C9—C16—H16B	110.9
C3—C4—H4B	108.8	C12—C16—H16B	110.9
C5—C4—H4B	108.8	H16A—C16—H16B	108.9
H4A—C4—H4B	107.7	O3—C17—O2	121.7 (4)
C19—C5—C4	109.0 (3)	O3—C17—C1	124.1 (4)
C19—C5—C15	111.9 (3)	O2—C17—C1	114.1 (4)
C4—C5—C15	107.7 (3)	C1—C18—H18A	109.5
C19—C5—C6	112.8 (3)	C1—C18—H18B	109.5
C4—C5—C6	107.3 (3)	H18A—C18—H18B	109.5
C15—C5—C6	107.9 (3)	C1—C18—H18C	109.5
C7—C6—C12	109.4 (3)	H18A—C18—H18C	109.5
C7—C6—C5	113.8 (3)	H18B—C18—H18C	109.5
C12—C6—C5	116.3 (3)	C5—C19—H19A	109.5
C7—C6—H6	105.5	C5—C19—H19B	109.5
C12—C6—H6	105.5	H19A—C19—H19B	109.5
C5—C6—H6	105.5	C5—C19—H19C	109.5
C8—C7—C6	113.4 (4)	H19A—C19—H19C	109.5
C8—C7—H7A	108.9	H19B—C19—H19C	109.5
C6—C7—H7A	108.9	C9—C20—H20A	109.5
C8—C7—H7B	108.9	C9—C20—H20B	109.5
C6—C7—H7B	108.9	H20A—C20—H20B	109.5
H7A—C7—H7B	107.7	C9—C20—H20C	109.5
C7—C8—C9	114.1 (3)	H20A—C20—H20C	109.5
C7—C8—H8A	108.7	H20B—C20—H20C	109.5
C9—C8—H8A	108.7	O2—C21—C22	108.3 (4)
C7—C8—H8B	108.7	O2—C21—H21A	110.0
C9—C8—H8B	108.7	C22—C21—H21A	110.0
H8A—C8—H8B	107.6	O2—C21—H21B	110.0
C16—C9—C20	116.3 (4)	C22—C21—H21B	110.0
C16—C9—C10	99.6 (3)	H21A—C21—H21B	108.4
C20—C9—C10	113.8 (3)	C21—C22—C23	114.8 (4)
C16—C9—C8	107.2 (3)	C21—C22—H22A	108.6
C20—C9—C8	111.5 (4)	C23—C22—H22A	108.6
C10—C9—C8	107.5 (4)	C21—C22—H22B	108.6
O1—C10—C11	126.1 (4)	C23—C22—H22B	108.6
O1—C10—C9	124.6 (4)	H22A—C22—H22B	107.5
C11—C10—C9	109.2 (4)	C24—C23—C22	113.5 (4)
C10—C11—C12	106.0 (3)	C24—C23—H23A	108.9
C10—C11—H11A	110.5	C22—C23—H23A	108.9
C12—C11—H11A	110.5	C24—C23—H23B	108.9
C10—C11—H11B	110.5	C22—C23—H23B	108.9
C12—C11—H11B	110.5	H23A—C23—H23B	107.7
H11A—C11—H11B	108.7	C23—C24—Br1	112.2 (4)
C13—C12—C16	110.4 (3)	C23—C24—H24A	109.2
C13—C12—C11	114.1 (3)	Br1—C24—H24A	109.2
C16—C12—C11	99.6 (3)	C23—C24—H24B	109.2
C13—C12—C6	111.3 (3)	Br1—C24—H24B	109.2
C16—C12—C6	107.3 (3)	H24A—C24—H24B	107.9
C11—C12—C6	113.2 (3)		
